# On Chloroform and the Narcotic Remedial Series

**Published:** 1861-05

**Authors:** 


On Chloroform and the Narcotic Remedial Series,—their Action and Application.—*Lecture XI.*—I have said that all the narcotic series have but one mode of action in their destruction of sensibility. I may add that this action is not altogether obscure; indeed it is not going too far to say that the *modus operandi* of these remedies is better known than that of any other class. We are indebted to Dr. Snow for the insight we have thus obtained. By a singularly happy series of experiments, Snow has proved that all the volatile narcotics have one principle in common: they arrest oxidation, and in such way stop the combustion of combustible bodies. Here is an experiment in point. I diffuse through this glass chamber chloroform vapor to the extent say of five per cent. of chloroform to the air contained. Giving a little time for diffusion, I now place in this chamber a lighted taper, and as you observe, the taper at once goes out. I repeat a similar experiment with ether, and although ether is an inflammable body when mixed with air, yet if I am adroit in the manipulation, the result is the same. I do it once more with amylene, the result is identical.

I might change the experiment a little for the purpose of illustration. I might take a portion of phosphorus, scrape it and expose it to the air. Under these circumstances I should see the surface undergo change; the phosphorus is absorbing oxygen gas, and it becomes coated with a thick white layer of oxide of phosphorus. I take another piece of phosphorus, scrape it in the same way, and expose it to air containing chloroform, and now I find that this process of oxidation does not occur.

Once more, I take a portion of fresh animal fibre, I expose this to the air and it undergoes change, putrefaction. I place another portion of animal fibre in air charged with chloroform and no such putrefaction is set up. Here, as a telling example, is a specimen of lung which was shown, thus preserved, at the Medical Society of London in 1851. It has never been removed from the bottle, and except that it has become slightly drier than it was originally, it presents no change.

The reason why chloroform and its analogues exert this arresting influence is not as yet scientifically explained.

The chloroform seems to undergo no change, and the oxygen seems to undergo no change. In our ignorance, therefore, of what absolutely occurs, we have invented a term for the fact—we say that the action is catalytic, or is due to catalysis.

Any way we have the fact, and it supplies us with a glance as to the action of the narcotics on man. I must explain in what manner.

The oxygen taken into the lungs in respiration is carried round the economy, and is applied to the oxidation of the oxidizable materials of the tissues. By this process of oxidation, ever persistent during life, new tissues are elaborated, old tissues are removed. By the same process animal heat is generated, and on the continuance of the process sensation, volition, and all the organic functions depend. When, therefore, chloroform or any other narcotic of the same kind is received into the lungs by the respiration, the chloroform is absorbed into the blood and is borne throughout the whole of the system. Thus carried, according to the theory of Dr. Snow, it arrests the process of oxidation, and therewith the functions of volition and sensation.

Carried far enough, then, the chloroform would cause absolute death. But withdrawn at the proper moment, its volatility saves that ultimatum. The vapor escapes from the body rapidly, and in proportion to its elimination, the arrested functions are restored.

In the process of narcotization, certain symptoms are produced in the animal body, which map out the action of the narcotic, and especially of chloroform, at different periods. Dr. Snow, whose authority we must again seek, divides these into four periods or degrees. In the first degree is included "all the effects of chloroform that exist while the patient retains a perfect consciousness of where he is, and what is occurring around him. This degree constitutes all that a person remembers of the effects of the vapor, except when he happens to dream, and recollects it afterwards."

In the second degree the consciousness is lost. "The mental functions," says Snow, "are impaired, but not necessarily suspended. The patient usually appears as if asleep in this degree, but if his eyelid be raised, he will move his eyes in a voluntary manner. There are occasionally voluntary movements of the limbs; and although the patient is generally silent, he may laugh, talk, or sing. Any dreams that the patient has occur while in this degree. The loss of sensation is now very considerable."

The third degree of narcotism is one of complete unconsciousness. Every movement made by the patient is involuntary. There is often in this degree rigidity and spasms of the muscles. The person in this state is incapable of "any perception of pain." In this stage almost all operations may be performed excepting those on the mouth, for the rigidity of muscle is frequently most marked in the muscles of the lower jaw, and the mouth is firmly closed.

In the fourth degree the breathing is stertorous, "the pupils are dilated, and the muscles are completely relaxed." The patient is always perfectly insensible.

The division of narcotization into these four stages is among the most exact of observed medical facts. Any one who has administered chloroform many times will at once recognize the truthfulness of the picture drawn. I shall show presently that the knowledge of these stages is essential in the administration of the narcotic.

The degrees of symptom thus observed in reference to chloroform extend in a general sense to the administration of ether and amylene. In etherization, however, the first degree is very much prolonged, the second is more boisterous, the third is more evanescent, and the fourth is more decidedly stertorous. Taking them altogether, the fourth degree of narcotism may be arrived at under chloroform more quickly than the second by ether. This, even when the chloroform is given deliberately and carefully.

In the administration of the narcotics, and especially of chloroform, certain points deserve consideration in detail. They may not appear of importance as they are noticed individually; but in the mass they supply the principles upon which the safe administration of the agent may be conducted.

In the first place, before ever chloroform is given to a patient, the exact physical condition of that patient should be ascertained. On this point I am and have always been at variance with an opinion of my late friend, Dr. Snow. Dr. Snow held that in all cases where an operation was demanded it was safe practice to give the chloroform, whatever might be the physical condition of the patient. He carried this rule to such an extreme that, in the majority of his cases, he never examined the person at all, and it is fair to admit that his rule was so far sound that out of four thousand administrations of the vapor, he met with but one fatal result. Nay, it is but fair further to admit that in instances where large and painful operations are contemplated, the determination as to the operation affirmatively is sufficient to determine the propriety of administering the narcotic.

But in trifling operations, such as tooth extractions and removal of tumors, in operations, I mean, where the danger to life is not present from the operation, the rule, in my opinion, entirely breaks down.

In ascertaining, then, the physical condition of the patient two important inquiries have to be made. 1st—Is the respiration healthy? Secondly—Is the heart healthy? These two inquiries must be conducted by means of physical diagnosis, *i. e.*, by the stethoscope, and by careful exploration of the chest, and I maintain, as I shall afterwards show by incidental remark, that no man is truly competent to the administration of the narcotic series who is not competent in this matter of diagnostic skill.

I lay it down as a rule, in short, which experience will every day strengthen, that the danger of chloroform is less in the mode of administration (though that is of no mean consideration) than in the exclusion of patients who could not take it without some risk more than ordinary to life.

The conditions under which chloroform becomes a dangerous remedy are of six kinds:—1. Cases of tubercle of the lung; 2. cases where there is irregularity of the heart from feebleness of the contractile muscular wall; 3. cases where the heart is very feeble, and the patient is loaded with fat; 4. cases where there is intense anæmia or bloodlessness; 5. cases where there is distinct disease of the kidney, attended with the secretion of albumen; 6. cases in which there is marked evidence of congestion or softening of the brain. There may be many more exceptions, but these occur to me as most prevalent and determinate.

It is no intention of mine to lay it down dogmatically, that in all these conditions chloroform is to be considered as strictly prohibited. Each case must be judged by the phenomena it presents, but in every case such as I have glanced at, chloroform is unadvisable in all small operations; in other words, chloroform in such examples is only advisable when it adds to the chances of success in the operation itself.

Granting that chloroform, or ether, has been decided on, it is well, wherever it is possible, to prepare the patient for it. It is best that the stomach should not be empty, and it is necessary, to avoid painful vomiting, that the stomach be not loaded at the time of the inhalation. A light meal with but little fluid, taken about two hours beforehand, is a good practice. Some practitioners whom I meet like to give the patient a glass of wine previous to the inhalation; there is no objection to this, and if there is any sign of faintness or fear, it is useful to give the wine, but the quantity must be limited to two ordinary wineglassfuls, otherwise the vomiting is increased and prolonged.

The position of the patient is another matter to be considered. It is, in my opinion, of little moment whether the sitting or recumbent position be chosen, but this I am convinced is of consequence, to let the patient retain the selected position for three or four minutes before inhalation. It is a curious physiological fact that the circulation changes in time on change of position. The pulse which was at seventy when a person is standing will fall to sixty-five, and I have known it fall to sixty on the same person lying down at full length. The pulse at seventy in the sitting position will fall to sixty-seven or sixty-five on the recumbent position being taken. The rise in the pulse in changing positions in the reverse way is equally well marked. The cause of this variation yet awaits explanation; but the variation is a fact, and as it is of moment to have the circulation as steady as possible during the administration of the narcotic, the patient should not only be kept in one posture for a short time previous to inhalation, but during the whole period of narcotization. If there is an exception to this rule, it is in case of syncope, then if the person is sitting, it may be necessary to bring the body slowly down to the horizontal posture.

Lastly, an effort should always be made to quiet the apprehension of the patient. A few gentle words, or a brief conversation enticing the mind to the consideration of other subjects than danger, or chance of danger, is invariably a wise and safe policy.

The administration of chloroform being agreed on, the next point to be considered is the regulation of the amount to be supplied. This can only be secured by the use of a proper inhaler. To give chloroform on a linen rag, a piece of lint, or a sponge, is at once as unscientific as it is wasteful, and as wasteful as it is unsafe. Given in this way, no check whatever is put upon the quantity absorbed by the patient, while the surrounding air, charged also with the vapor, is annoying to the operator and to all around. I have seen a bystander obliged to leave the operating table owing to the influence of the chloroform vapor, all of which was being wasted.

The best form of inhaler is Snow's, with Sibson's mouthpiece. I will pass it round that you may see its construction. There are in it two chambers; one (the outer) for holding water to secure an equality of temperature; the other (the inner) for holding the chloroform.

Down the center of the inner chamber there passes a slight frame-work covered with bibulous paper, upon which the chloroform falls when it is poured into the inhaler. Air to dilute the narcotic is freely admitted by a series of holes opening into the inner chamber, while the exit opening is large, and communicates with an elastic tube one inch in diameter, and six or eight inches long, for connection with the mouthpiece.

The mouthpiece, made of thin metal, is double valved. One valve, directed inwards, prevents the breath exhaled by the patient from reëntering the inhaler. The outer valve secures the escape of the expired air, and being under the control of the administrator of the chloroform, serves the important purpose of enabling him to admit a larger volume of air, when that is required.

The amount of chloroform required is governed almost by mathematical law, when the inhaler above named is used. In commencing the inhalation, one drachm and a half of chloroform is the best quantity to put in. This will ordinarily suffice to carry an adult patient into the third degree of narcotism. It is well to commence with the exit valve altogether turned off, by which means a large volume of air is inspired, and the patient is accustomed to inhale the vapor comfortably; after half a minute or so, the valve may be turned on, but it need rarely be carried entirely over the opening. In the majority of cases, the necessary degree of narcotism for any quick operation is thus secured; but if the operation is prolonged, more chloroform may be added in half-drachm doses.

The degree to which the insensibility should be carried varies with the kind of operation. In tooth extraction, it is very often not necessary to pass beyond the second degree. This is particularly the case where only one tooth has to be removed; but inasmuch as the chloroform has to be withdrawn when the operation is being performed, it will be found always best practice, when several extractions have to be made or tedious operations to be done, to carry the narcotism to the third degree, and if there is much rigidity in the muscles of the jaw, so that the mouth can not be opened, to the fourth degree. When the eyes are rolled upwards and the muscles are relaxed, the operation may safely begin, nor is there any fear of a return of sensation for the space of at least four minutes. The patient may move a little or flinch, but these actions are not produced by pain.

As soon as an operation is concluded, the patient should be freely supplied with air; and he is much better let alone until consciousness is complete. If vomiting occurs, it is, in my opinion, best to encourage it, for in the vomited matter chloroform is thrown off. The stomach *i. e.* takes on eliminatory functions, and the sooner the chloroform is expelled, the sooner the vomiting ceases.

The causes of danger and of death during the inhalation of chloroform, and indeed of all the volatile narcotics, rest upon their action on one of three parts—the brain, the heart, or the lungs. Different narcotics influence these parts differently. In poisoning from ether, in so far as I can judge from experiments on inferior animals, the effect is commenced in the brain. The fumes of the lycoperdon seem first to act on the respiration, checking that function. The vapor of chloroform again appears primarily to produce a depressing influence on the heart. In nearly every fatal case after chloroform, the report goes, "the pulse suddenly stopped." The death, therefore, is by syncope. This view is supported by experiment. If the chest of an animal be opened, and the respiration be sustained by artificial means, the heart will continue beating for a long period of time, and its contractions and relaxations may be carefully observed. Now, in this state, if a little vapor of chloroform is blown upon the pulsating organ, the action will stop and the heart will remain paralyzed until such time as the chloroform driven upon it has been removed either by evaporation or absorption. When the removal occurs, then the heart once more starts off into active play.

If in an experiment of this nature, the chloroform, instead of being blown upon the heart, be injected into the great aorta at its commencement so as to feed the coronary circulation, or, in other words, the heart itself, with the narcotic, the same result, arrest in the contraction of the organ, will ensue.

I am speaking now from direct experiments, personally observed; we gather from them, as well as from the symptoms in cases of fatal inhalation, the fact, that chloroform is fatal by an immediate influence upon the heart, a fact which Dr. Sibson was the first to suggest.

After death from the narcotics, certain *post mortem* appearances have been observed, which require notice. In the human subject the effects of ether have not been much recognized, owing to the lucky circumstance that there has only been one certain death. In animals killed by ether, there is produced general congestion of the lungs of the right side of the heart and of the brain. The whole of the soft structures as well as the blood evolve an ethereal odor, and the blood is sometimes more fluid than ordinary. After death from chloroform, and the remarks in a general way extend also to amylene, the lungs are not specially congested. Indeed, in the inspection of nearly two hundred animals killed by chloroform, I have never once found the lungs congested. I have rather found them pale and bloodless, in some instances quite white. Dr. Snow records a similar experience. In the human subject, however, in several cases the lungs have been found somewhat congested. The reason of this difference, granting it to exist, is not very plain. In all cases, human and comparative, the right side of the heart and the great vessels are found engorged with blood, while the left side is usually quite empty; conditions, each alike indicating that the primary failure or paralysis is in the central organ of the circulation. The blood after death by chloroform is sometimes quite fluid, but inasmuch as it coagulates on exposure to the air, its fluidity must be considered as dependent on its confinement in the vessels, and on the presence of the narcotic vapor. This view is further affirmed in the circumstance that the addition of chloroform to newly drawn blood does not materially interfere with the process of coagulation. No special modification of the nervous system has been observed after death from the narcotics named.

The conditions which seem to have favored the occurrence of death from chloroform have been much commented on by various authors. It has been noticed as a peculiar fact that in a great number of fatal cases, the death has happened either before the operation, or during an operation very trivial in its nature. Hence a conclusion has been drawn that a large operation is favorable to the success of chloroformization as a process. Dr. Snow was not opposed to this hypothesis; on the contrary, he was inclined to think that the loss of a moderate quantity of blood was in some instances of advantage. For my own part, I can find no data of sufficient extent to guide me to a conclusion on this point.

Diseases of the lungs is a second condition which has been supposed to contribute to a fatal catastrophe from chloroform. In a few of these cases, tubercle of the lung has been found as a preëxistent malady, and so found has had possibly a share in the result.

But the diseased states which most of all are causes of death, are those in which the heart is implicated; the condition of heart most dangerous is that in which *the walls* of the organ, from structural change, have lost, more or less, their power of contractility. I look upon this form of disease as even more important than valvular disease, always supposing that the obstruction arising from diseased valves is unattended with symptoms of immediate danger. To stay to point out the indications of softening of the walls of the heart would lead me into an argument too long for the present time, and too purely medical to be strictly in place here. I must therefore rest satisfied with the above general statement. You will ask me what is the best treatment to be adopted in cases where during the administration of a narcotic vapor, and especially of chloroform, danger presses. The treatment is summed up in a few words. If the patient is breathing, at once withdraw the narcotic and give plenty of fresh air; if the patient is not breathing at once, do not hesitate an instant, but set up artificial respiration. This may be done either by mouth to mouth inflation, or by Dr. Marshall Hall's plan of placing the patient on the face, pressing the back, then bringing him on to the side, and repeating these steps some fifteen or twenty times a minute; or, lastly, by the use of a pair of double acting bellows, such as I now place before you. These bellows, which may be carried in the coat pocket, have this advantage: in expansion they empty the lungs of the air contained, and in closure they fill the lungs with new air from the atmosphere. In inferior animals I can restart the heart, after it has ceased to beat, by these bellows, but no time is to be lost, a resting heart will continue in a condition to recover but a few seconds at most.

I have been asked to express an opinion as to local anæsthesia in dental operations. This is soon done, for I may affirm that there is no known local anæsthetic which is at once certain, safe, and practical for operations in the mouth. Congelation is often effective, but is not convenient. The external application of a narcotic is in most cases, and in the cases best adapted to it, but partially successful. The electrical current, about which we have heard so much, is no anæsthetic whatever. It may divert sensation, it never conquers pain.

I have lately tried to introduce a new mode of producing insensibility locally by using a narcotic combined with the continuous electrical current. This process, which in some instances has been very successful, and in other instances equally unsuccessful in causing insensibility of the soft structures, is inappropriate as a general measure in cases of tooth extraction. It has been successful truly in a few cases of this kind, but it is only of service where the pulp cavity is laid open ; for the hard tooth structure being a non-conductor of the electrical current, and a bad absorbing surface, no influence is produced at all on the sensitive structure if these be enclosed in dentine and enamel.

I do not, therefore, press this method of operation on your notice, for it is a poor ambition to push forward an indifferent system because it has been suggested by oneself. Moreover, I hope at another time to show you some steps towards improvement, and indeed to bring forward the subject of anæsthesia altogether on a much more comprehensive scale.—*Dr. Richardson's Lectures, reported for the Dental Review.*

